# Draft Genome Sequence of Enterococcus dispar CoE-457-22, Isolated from Traditionally Produced Montenegrin Dry Sausage

**DOI:** 10.1128/mra.01038-22

**Published:** 2022-12-14

**Authors:** Beatriz Daza Prieto, Nadja Raicevic, Johann Ladstätter, Patrick Hyden, Ana Jovanovic, Anna Stöger, Adriana Cabal Rosel, Robert L. Mach, Werner Ruppitsch, Aleksandra Martinovic

**Affiliations:** a Institute of Medical Microbiology and Hygiene, Austrian Agency for Health and Food Safety, Vienna, Austria; b Centre of Excellence for Digitalization of Microbial Food Safety Risk Assessment and Quality Parameters for Accurate Food Authenticity Certification (FoodHub), University of Donja Gorica, Podgorica, Montenegro; c Technical University of Vienna, Institute of Chemical, Environmental, and Bioscience Engineering, Research Area of Biochemical Technology, Vienna, Austria; d Department of Biotechnology, University of Natural Resources and Life Sciences, Vienna, Austria; Indiana University, Bloomington

## Abstract

Enterococcus dispar was isolated for the first time from synovial fluid and stool cultures and described as a new species in 1991. Here, we report the genome of E. dispar CoE-457-22, which was obtained from traditionally produced Montenegrin dry sausage (sudzuk).

## ANNOUNCEMENT

Limited information is available about Enterococcus dispar. Similar to other enterococci, E. dispar has been isolated from the environment (1, 2), animals (3), food (4), and clinical samples (5–8). Genomic analysis of strain CoE-457-22, which was isolated from Montenegrin dry sausage, may provide useful information on environmental adaptation, safety aspects (9), and contributions to the typicity of traditional food products (10).

Sample enrichment and isolation of bacterial strains from traditional Montenegrin dry sausage (sudzuk) were performed using de Man, Rogosa, and Sharpe (MRS) agar (Thermo Fisher Scientific, Oxoid Deutschland) according to the ISO 15214:1998 method (11). Colonies that were morphologically suspected to be lactic acid bacteria (LAB) were subcultured on MRS agar for species identification by matrix-assisted laser desorption ionization–time of flight mass spectrometry (MALDI-TOF MS) (Microflex LT/SH system with the MBT Compass IVD 4.1.100 module; Bruker, Billerica, MA, USA) and whole-genome sequencing (WGS).

Genomic DNA was isolated from overnight cultures grown on blood agar at 37°C using the MagAttract high-molecular-weight (HMW) DNA kit (Qiagen, Hilden, Germany). Libraries were prepared using a DNA preparation (M) kit (Illumina, Inc., San Diego, CA, USA), and 2 × 150-bp sequencing was performed on a NextSeq 2000 instrument (Illumina, Inc.) as described previously (12). A long-read sequencing library was prepared using the rapid barcoding sequencing kit (SQK-RBK004; Oxford Nanopore Technologies, Oxford, UK) and sequenced with a FLO-MIN106D R9.4.1 SpotON flow cell on a MinION Mk1C device, according to the manufacturer’s instructions. A total of 6,841 Nanopore reads, with an *N*_50_ value of 5,883 bp, were obtained using guppy v6.1.5 in fast base-calling mode and filtered using Filtlong v0.2.1 with the following parameters: min_length, 1000; keep_percent, 90; target_bases, 500000000. A total of 5,349,760 Illumina reads were quality controlled using FastQC v0.11.9 and used untrimmed in the final hybrid assembly using Unicycler v0.5.0, which resulted in 6 contigs with a mean coverage of 287-fold, an *N*_50_ value of 2,293,182 bp, a genome size of 2.7 Mb, and a GC content of 37.2% (Table 1).

**TABLE 1:** Characteristics of Enterococcus dispar CoE-457-22

Attribute	Finding
Assembly size (bp)	2,662,631
Avg contig length (bp)	443,771
No. of contigs	6
*N*_50_ (bp)	2,293,182
GC content (%)	37.2
Genome coverage (×)	287
Total no. of genes	2,604
Total no. of coding sequences	2,537
No. of RNA genes	67
No. of noncoding RNA genes	4
No. of tRNAs	60
No. of CRISPR/Cas elements	1
Pathogenic families	Hypothetical protein (GenBank accession no. BAF77294, BAF77275, and BAF77283), transmembrane amino acid transporter protein (GenBank accession no. CBJ22574), conserved hypothetical protein (GenBank accession no. CBJ22562, CBJ22569, CBJ22565, and CBJ22571), small subunit ribosomal protein S19P (GenBank accession no. ABP89047), 30S ribosomal protein S21 (GenBank accession no. ABP90399), polyribonucleotide nucleotidyltransferase (GenBank accession no. CBJ22582), and ribosomal protein L29 (GenBank accession no. AA080083)

The Comprehensive Antibiotic Resistance Database (CARD) and tools from the Center for Genomic Epidemiology (http://www.genomicepidemiology.org) were used for detection of antimicrobial resistance genes (ARGs), virulence genes (VGs), plasmids, and pathogen families. BAGEL4 (13) and antiSMASH v6.0 (14) were used to detect genes for the synthesis of secondary metabolites and aromatic compounds. Default parameters were used for all software unless otherwise specified. Vancomycin and tetracycline susceptibility testing was performed using Etest strips (bioMérieux, Vienna, Austria).

The NCBI Prokaryotic Genome Annotation Pipeline (PGAP) (15) identified 2,604 genes, 2,537 coding sequences, 19 pseudogenes, and 67 RNA genes (Table 1). Basic Local Alignment Search Tool (BLAST) (16) analysis revealed 99.56% similarity of the 16S rRNA genes between CoE-457-22 and E. dispar ATCC 21566. Digital DNA-DNA hybridization (dDDH) (formula d4) (https://tygs.dsmz.de) and average nucleotide identity (ANI) (17) analyses revealed values of 94.8% and 98.99%, respectively, for similarity between CoE-457-22 and E. dispar ATCC 51266.

CoE-457-22 carried the ARGs *tetM*, *vanY*, and *vanT* and was sensitive to vancomycin and resistant to tetracycline. Plasmids, VGs, bacteriocin genes, and secondary metabolite biosynthetic gene clusters were not detected. PathogenFinder identified 12 pathogenic families (18) (Table 1).

### Data availability.

This whole-genome shotgun project has been deposited in DDBJ/ENA/GenBank under BioProject accession number PRJNA882413 and BioSample accession number SAMN30933858. This is the first version of this genome. The raw sequence reads have been deposited in the Sequence Read Archive (SRA) under the accession numbers SRR21656176 and SRR22245459.
